# Personal

**Published:** 1895-01

**Authors:** 


### Personal.

DR. EDMUND M. POND has opened a private hospital at No. 20 Nichols street, Rutland, Vt. Miss Janet R. S. Sheldon is associated with Dr. Pond in the conduct of the hospital.

The building, constructed but a few years ago at a cost of $30,000, is beautifully situated and is surrounded by three acres of land. Large, airy wards and private rooms are newly furnished and will accommodate thirty-five patients. A modern aseptic operating room, with improved tables, sterilizers and instruments, is connected with the hospital. Trained hospital nurses are in constant attendance, and, if desired, can be furnished for cases outside of the hospital. One charity case at a time will be taken free of all charges.

---

DR. WILLIAM P. SPRATLING, a graduate of the College of Physicians and Surgeons of Baltimore of the class of 1886, according to the *Maryland Medical Journal*, has been elected superintendent of the Craig Colony for Epileptics, in the State of New York. Dr. Spratling will visit the famous Colony for Epileptics at Bielefeld, Germany, before entering upon his duties at Craig Colony.

Dr. E. J. Gilray, of Buffalo, has been appointed house surgeon and superintendent of the Erie County Hospital, vice Dr. J. Haberstro, resigned.

Dr. Gilray was graduated from the University of Buffalo five years ago. He confined himself to hospital work for several years He was chief medical interne at the Harper Hospital in Detroit for some time.

---

Dr. Willis P. King, of Kansas City, who has been in poor health for some months, is reported to be improving. He will spend the winter in Southern Texas, in the full expectation that prolonged rest will restore him to his wonted vigor. We join his many friends in the hope that this pleasant prospect may be fully realized.

---

Dr. Henry Y. Grant, of Buffalo, was elected a member of the Ophthalmological Society of the United Kingdom of Great Britain and Ireland at a recent meeting. This is a merited distinction conferred upon our distinguished townsman, who is one of our eminent ophthalmologists.

---

Dr. J. B. Murphy, of Chicago, has announced to the medical profession that henceforth his practice will be limited to surgery, operative gynecology and consultations. His offices are in the Venetian building.

---

Dr. Clinton Cushing, of San Francisco, spent Thanksgiving Day in Buffalo, en route to New York, and thence to Europe. He was the guest of Dr. and Mrs. William Warren Potter, of Franklin street.

